# Impact of sepsis on older and nonolder patients: clinical conditions and outcomes

**DOI:** 10.1186/cc12953

**Published:** 2013-11-05

**Authors:** Jaqueline Lima de Souza, Fábio Ferreira Amorim, Adriell Ramalho Santana, Felipe Bozi Soares, Bárbara Magalhães Menezes, Mariana Pinheiro Barbosa de Araújo, Fernanda Vilas Bôas Araújo, Louise Cristhine de Carvalho Santos, Pedro Henrique Gomes Rocha, Alessandra Vasconcelos da Silva Paiva, Gabriel Kanhouche, Pedro Nery Ferreira Júnior, Alethea Patrícia Pontes Amorim, José Aires de Araújo Neto, Edmilson Bastos de Moura, Marcelo de Oliveira Maia

**Affiliations:** 1 Escola Superior de Ciências da Saúde, Brasília, Brazil; 2 Liga Acadêmica de Medicina Intensiva de Brasília, Brazil; 3 Hospital Santa Luzia, Brasília, Brazil

## Background

The increase of the older population, with higher incidence of co-morbidities and age-related decline in organic functions, suggests a need for better understanding the peculiarities of the process leading to adequate critical care in this group of patients [1,2]. This study aims to evaluate morbimortality of sepsis on older and nonolder patients and its impact on their outcomes.

## Materials and methods

Retrospective cohort study conducted in the ICU of Hospital Santa Luzia, Brasilia, DF, Brazil, during 6 months. Patients diagnosed with sepsis were divided into two groups: older, defined as age ≥65 years, and nonolder, with age <65, for the analysis of the outcomes.

## Results

A total of 130 patients with sepsis were enrolled, 11.5% (*n *= 15) with septic shock. Mean age was 64 ± 22 years, ICU length of stay 9.6 ± 13.2 days, SAPS3 50 ± 13. ICU mortality in 4 days was 6.9% (*n *= 13), in 28 days was 9.2% (*n *= 12) and hospital morality was 17.7% (*n *= 23). Seventy patients were older (60.8%). The older patients had higher SAPS3 (56 ± 12 vs. 40 ± 9, *P *= 0.00), Charlson Comorbidity Index (CCI) (2.4 ± 1.9 vs. 1.0 ± 1.7, *P *= 0.00), and incidence of Glasgow Coma Scale <15 (19% vs. 5.9%, *P *= 0.03). There was no difference between the groups regarding the incidence of septic shock (12.7% vs. 9.8%, *P *= 0.62), need for dialysis (8.9% vs. 7.8%, *P *= 0.84), vasopressor agents (6.3% vs. 7.8%, *P *= 0.74) or invasive mechanical ventilation (25.3% vs. 15.7%, *P *= 0.19). There was also no difference regarding ICU length of stay (15 ± 2 vs. 10 ± 1 days, *P *= 0.22), mortality in 4 days (8.9% vs. 3.9%, *P *= 0.28) and in 28 days (12.7% vs. 3.9%, *P *= 0.09). However, hospital mortality was higher amongst the group of older patients (26.5% vs. 3.9%, *P *= 0.00). The relative risk of hospital death in older patients was 6.78 (95% CI: 1.66 to 27.68).

## Conclusions

Older septic patients had higher SAPS 3, CCI, and incidence of GCS <15. Although there was no difference regarding mortality in 4 and 28 days or ICU length of stay, hospital mortality was higher for this group of patients.
